# Differential expression of Toll-like receptors and inflammatory cytokines in ovine interdigital dermatitis and footrot

**DOI:** 10.1016/j.vetimm.2014.07.007

**Published:** 2014-09-15

**Authors:** Rebecca Davenport, Christopher Heawood, Kate Sessford, Melissa Baker, Kerstin Baiker, Barbara Blacklaws, Jasmeet Kaler, Laura Green, Sabine Tötemeyer

**Affiliations:** a School of Veterinary Medicine and Science, University of Nottingham, Loughborough LE12 5RD, UK; b Department of Veterinary Medicine, University of Cambridge, Madingley Road, Cambridge CB3 0ES, UK; c School of Life Sciences, University of Warwick, Coventry CV4 7AL, UK

**Keywords:** Footrot, Sheep, Toll-like receptor, Inflammation, Histopathology, Cytokines

## Abstract

Footrot is a common inflammatory bacterial disease affecting the health and welfare of sheep worldwide. The pathogenesis of footrot is complex and multifactorial. The primary causal pathogen is the anaerobic bacterium *Dichelobacter nodosus*, with *Fusobacterium necrophorum* also shown to play a key role in disease. Since immune-mediated pathology is implicated, the aim of this research was to investigate the role of the host response in interdigital dermatitis (ID) and footrot. We compared the expression of Toll-like receptors (TLRs) and pro-inflammatory cytokines and the histological appearance of clinically normal in comparison to ID and footrot affected tissues. Severe ID and footrot were characterised by significantly increased transcript levels of pro-inflammatory cytokines TNFα and IL1β and the pattern recognition receptors TLR2 and TLR4 in the interdigital skin. This was reflected in the histopathological appearance, with ID and footrot presenting progressive chronic-active pododermatitis with a mixed lymphocytic and neutrophilic infiltration, gradually increasing from a mild form in clinically normal feet, to moderate in ID and to a focally severe form with frequent areas of purulence in footrot. Stimulation with *F. necrophorum* and/or *D. nodosus* extracts demonstrated that dermal fibroblasts, the resident cell type of the dermis, also contribute to the inflammatory response to footrot bacteria by increased expression of TNFα, IL1β and TLR2. Overall, ID and footrot lead to a local inflammatory response given that expression levels of TLRs and IL1β were dependent on the disease state of the foot not the animal.

## 1 Introduction

Footrot is a bacterial infection of the interdigital skin of the sheep foot resulting in lameness, and is the greatest welfare and economic concern for sheep farmers and veterinary surgeons in the UK (Goddard et al., 2006). In England, more than 95% of sheep flocks have footrot (Kaler and Green, 2008). In the UK current vaccination strategies, while recommended as part of a comprehensive lameness management programme (EBLEX, 2013, FAI, 2014), are on their own neither long lasting nor efficacious (Duncan et al., 2012).

The pathogenesis of footrot is complex and multifactorial. Physical damage to the interdigital skin that is caused by, for example, prolonged exposure to moisture is required to initiate disease. Bacterial replication in this damaged skin leads to interdigital dermatitis (ID) where the superficial epidermal layers are inflamed, damaged and slough off irregularly. Disease may progress to footrot with separation of the hoof horn capsule from the underlying sensitive tissue. Hoof horn separation does not occur without the involvement of *Dichelobacter nodosus*, an obligate anaerobe bacterium. A second bacterium, *Fusobacterium necrophorum*, may also play a role in the pathogenesis of footrot. It either facilitates disease development by increasing the damage to the interdigital skin and promoting ID that subsequently permits replication of *D. nodosus* (Egerton et al., 1969, Roberts and Egerton, 1969) or *F. necrophorum* is secondary to *D. nodosus* but may exacerbate the severity and persistence of footrot (Beveridge, 1941, Kennan et al., 2010, Kennan et al., 2011, Witcomb et al., 2014).

We hypothesise that the pathology of footrot is a host mediated over-expression of local immune responses leading to acute severe inflammation in the foot that can progress to hoof horn separation from underlying tissues. The host response to bacterial invasion is characterised by the recruitment of large numbers of neutrophils into the epidermis. This causes inflammation and pressure in the hoof horn capsule resulting in its separation from the underlying tissue. However, not all sheep exposed to *D. nodosus* get footrot and fewer than half of cases progress to separation of the hoof horn (Wassink et al., 2010). Early histopathological observations of tissue sections from footrot lesions described little inflammatory response in areas with *D. nodosus*, but severe inflammation in response to invasion by *F. necrophorum* (Egerton et al., 1969). Infiltration by polymorphonuclear leucocytes and a dense population of filamentous bacteria, visually identified as *F. necrophorum* were observed in ID sections (Parsonson et al., 1967). It is, therefore, beneficial to elucidate the basis of the ovine inflammatory response in the interdigital skin to these pathogens and, thus, contribute to the more targeted development of novel vaccines and their adjuvants.

Key components of the host response to bacterial infection are the innate immune pathogen recognition receptors, such as the Toll-like receptors (TLRs) that are expressed in a wide range of cell types (Hancock et al., 2012). TLRs 1–10, which recognise a range of pathogen associated molecular patterns, have been cloned and characterised in sheep (Chang et al., 2009). Footrot is a mixed bacterial infection and, therefore, the TLRs likely to be involved in their recognition are TLR2, TLR2/TLR1 and TLR2/TLR6 heterodimers (which recognise bacterial lipoproteins, lipoteichoic acid and atypical LPS, respectively), TLR4 (which is activated by bacterial LPS), TLR5 (which recognises bacterial flagellin) and TLR9 (which recognises bacterial DNA). *D. nodosus* and *F. necrophorum* are both Gram-negative bacteria but, interestingly, *F. necrophorum* may have an atypical lipopolysaccharide (LPS) as shown for a closely related bacterium, *Fusobacterium nucleatum* (Kikkert et al., 2007) and hence may be signalling via TLR2. The activation of TLRs initiates a complex signalling network, leading to the expression of a wide range of inflammatory mediators such as nitric oxide, cytokines and chemokines (Carpenter and O’Neill, 2007, Takeda and Akira, 2004). Key pro-inflammatory cytokines of the skin include IL-1β and TNF-α (Nestle et al., 2009).

The aim of this research was to investigate this inflammatory response by focussing on histological appearance and the expression of TLRs and pro-inflammatory cytokines in clinically normal, ID and footrot tissues and cultured ovine dermal fibroblasts stimulated with bacterial extracts.

## 2 Materials and methods

### 2.1 Ovine interdigital skin biopsies

Ovine feet, obtained from an abattoir, were cleaned, scored for ID and footrot using a scoring system adapted from Parsonson et al. (1967) and classified into one of four categories; clinically healthy, mild ID (slight ID +/− foetid smell, <5% of area affected), severe ID (moderate − severe ID with a foetid smell, >5% of area affected) and footrot (active footrot lesion with under-running of the heel and/or sole area of the digit). Six millimetre biopsies from healthy and a range of severities of diseased feet were sampled from the skin/hoof interface and stored in RNAlater (Sigma, UK) for RNA isolation or fixed in 10% neutral buffered formalin (NBF).

### 2.2 Histopathology

For histopathology, the formaldehyde fixed biopsies (see above) from 10 normal, 7 ID and 8 footrot affected ovine feet were processed and embedded in paraffin wax. Upon wax embedding, biopsies were orientated to ensure a cross section of the skin-hoof interface was obtained. Cutting these wax blocks was facilitated by soaking the blocks in ice cold 10% ammoniated water prior to cutting. Sections (5 μm) were cut and stained with haematoxylin and eosin (H&E). Microscopy (Leica model DM5000B, software Leica 2000) was used to analyse the tissue sections.

### 2.3 Cell stimulation

Ovine fibroblasts from five individual Finn Dorset crossed sheep (B1123, 1378X, 1211A, 1220A and 1222A) were isolated and grown as described by Bird et al. (1993). For stimulation studies, they were cultured in Dulbecco's Modified Eagle's Medium supplemented with 10% foetal bovine serum, 2 mM l-glutamine, 5 μg/ml penicillin/streptomycin (Gibco), 0.63 μg/ml fungizone (Lonza) and 100 μg/ml gentamicin (Sigma). Cells were stimulated with *Escherichia coli* LPS (1 μg/ml) or heat killed *D. nodosus*, *F. necrophorum* or both (10 μg/ml) as established by preliminary experiments with 1–100 μg/ml bacterial extracts (data not shown). To capture the early host response, the cells were stimulated for 4 h (Widdison et al., 2011). For subsequent RNA isolation, cells were lysed with 350 μl of RNA lysis buffer (Nucleospin^®^ RNA isolation kits, Machery-Nagel, UK).

### 2.4 RNA isolation and cDNA synthesis

Biopsies were homogenised using a 5 mm stainless steel ball-bearing in a Retsch^®^ Bead Mill MM 301 for 4 min, at 30 oscillations per second. Total RNA from biopsy homogenate and lysed fibroblasts was isolated using Nucleospin^®^ RNA isolation kits (Machery-Nagel, UK) following the manufacturer's instructions. RNA concentration was measured using a NanoDrop™ (ND-1000, ThermoScientific, UK). RNA was diluted in water to 100 ng/μl, and cDNA synthesised using M-MLV Reverse Transcriptase and random hexamers (Promega, Madison, USA) according to manufacturer's instructions. To identify any residual genomic DNA contamination, samples with and without reverse transcription were PCR amplified for GAPDH, an abundant transcript, using forward (5′-CCACCAACTGCTTGGCCCCC-3′) and reverse (5′-GGACACGTTGGGGGTGGGGA-3′) primers. This was performed using DreamTaq Polymerase (Fermentas Life Sciences, York, UK), 10 μM of each primer, 10 μM dNTP mix (Promega, Madison, USA) and 25 ng of cDNA. The PCR program consisted of a 95 °C denaturation for 3 min, 25 cycles at 95 °C for 10 s, 55 °C for 1 min, 72 °C for 1 min, and a final extension at 72 °C for 10 min.

### 2.5 Quantitative real time PCR

All quantitative real time PCR (qPCR) experiments were designed and performed to comply with the quality controls detailed in the MIQE guidelines (Bustin et al., 2010). Primers for TLR2 (forward 5′-CATCTTTGTGCTTTCGGAGA-3′, reverse 5′-AAGAGACGGAAGTGGGAGAA-3′, 78 bp product, AM981300.1) and TLR4 (forward: 5′-AGAAACCTCCGCTACCTTGA, reverse: 5′-CAGGGAGCAAGTTGTTCTGA-3′, 130 bp, NM_001135930.1) were designed and assessed using NCBI PrimerBLAST. In addition qPCR primers for ovine β-actin, GAPDH (Hughes et al., 2011), PPIA (Lloyd et al., 2012), β2-microglobulin, α-tubulin, 18S, TLR1 (Taylor et al., 2008), TLR6 (Plain et al., 2010), TNFα (Sow et al., 2009) and IL-1β (Darlay et al., 2011) were used. The mRNA expression was measured using qPCR on a LightCycler^®^ 480 (Roche Applied Science, UK). Reactions contained 5 μl of diluted sample cDNA (1/10 dilutions apart from 18S and β-actin, where 1/100 dilution was used) in 1× SYBR green qPCR master mix (Roche Applied Science, UK) with 1 μM forward and reverse primers. The following cycle conditions were used: 95 °C for 10 min followed by 45 cycles at 95 °C for 10 s, 60 °C for 50 s, 72 °C for 1 min and a final dissociation gradient up to 97 °C to obtain a melt curve. Standard curves were generated using ovine lymph node or fibroblast cDNA to assess primer efficiency (Table S1).

Supplementary table related to this article found, in the online version, at http://dx.doi.org/10.1016/j.vetimm.2014.07.007.

Supplementary Table S1 Standard curve data from qPCR.

To facilitate mRNA expression studies, the six housekeeping genes were investigated to detemine a stable reference gene transcript. geNORM (Vandesompele et al., 2002) analysis for stability of expression was performed following qPCR analysis of biopsies from 16 clinically normal, eight ID, and four footrot ovine feet. Stability of a gene is defined as the consistancy of expression between samples. The geNorm recommended minimum (*M*) value for stability is 0.5. For normalisation, β-actin was used as the housekeeping gene (*M*-value of 0.49).

*C*_*t*_ values for each sample and transcript were calculated from the mean of triplicate reactions. Normalised expression of each gene was calculated using the following formulae (Hughes et al., 2007):

$$\text{Differentiation factor}=\frac{\text{overall mean of}\;40-\text{CP value for housekeeping gene}}{40-\text{CP value of housekeeping gene of thatsample}}$$

$$\text{Normalised expression}=\frac{\text{Mean}\;(40-\text{CP value for the sample})\times \text{target primer slope}}{\text{Differentiation factor for that sample}\times \text{housekeeping gene}}$$

Fold change = 2^(*T*−*C*)^, whereby *T* = normalised expression level of treated samples; *C* = s normalised expression level of control samples (Hughes et al., 2007). Expression data are presented as box and whisker plots showing minimum, lower quartile, median, upper quartile and maximum.

### 2.6 Statistical analysis

The biopsy data were modelled in a mixed effect two level model (Dohoo et al., 2009), which incorporated autocorrelation of feet within sheep. The model took the form:

$$Y_{ij}=\alpha +\beta_{1}X_{ij}+v_{j}+e_{ij}$$

$$v_{j}∼N(0,\Omega_{v}^{2})$$

$$e_{ij}∼N(0,\Omega_{e}^{2})$$

where *Y* is the immune response, the subscripts *i* and *j* denote the *i*th foot in the *j*th sheep respectively, *α* the regression intercept, *X*_*ij*_ the vector of covariates associated with each observation, *β*_1_ the coefficients for covariates *X*_*ij*_, *v*_*j*_ a random effect to reflect residual variation between sheep (mean = 0 and variance $\Omega_{v}^{2}$), *e*_*ij*_ a random effect to reflect residual variation between feet restricted to following a binomial distribution. For detailed model results with all confidence intervals (CI) see Supplementary Table S2.

Supplementary table related to this article found, in the online version, at http://dx.doi.org/10.1016/j.vetimm.2014.07.007.

Supplementary Table S2 Statistical model outputs – changes in expression in ID and footrot (FR) samples compared to clinically healthy samples.

The stimulated fibroblast data were analysed using One-way Analysis of Variance (ANOVA) with repeated measures followed by a Dunnett post-test.

## 3 Results

### 3.1 *TLR2*, *TLR4* and *IL-1β* expression was increased in severe ID and footrot

**Fig. 1:**
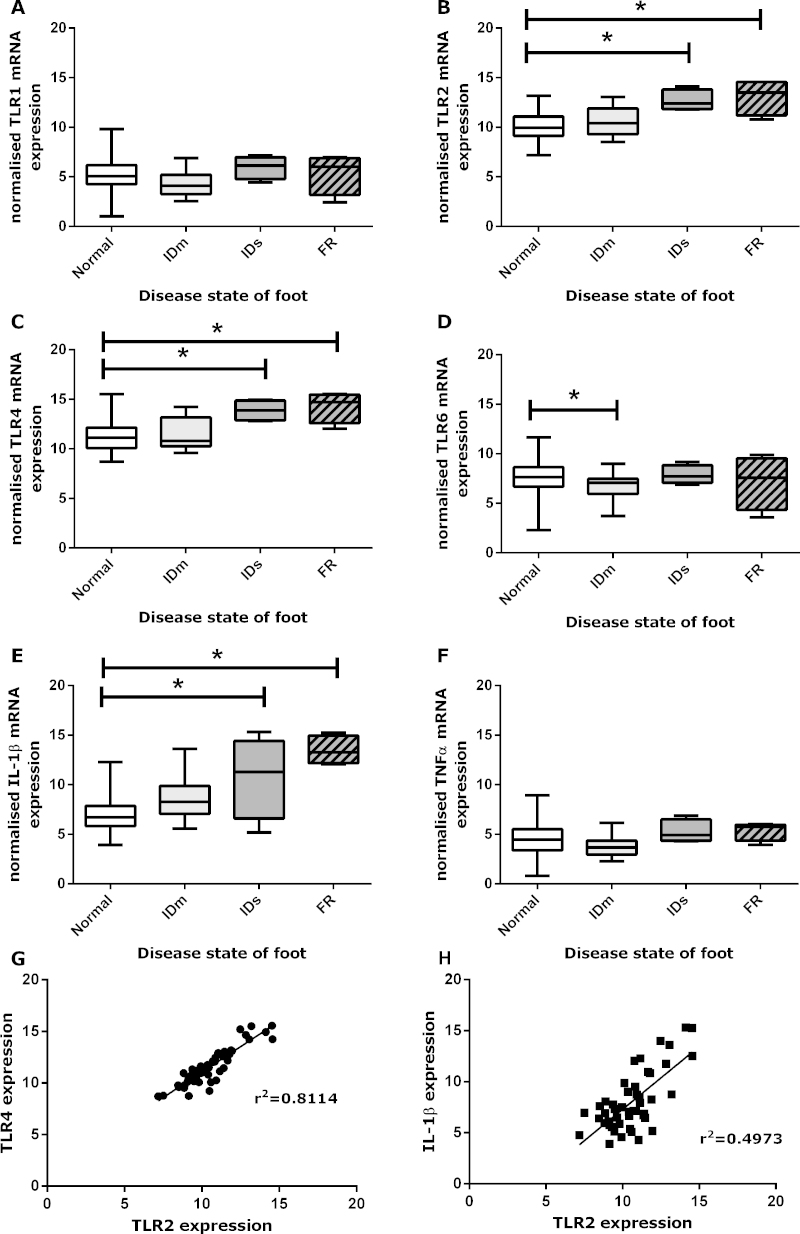
TLR and inflammatory mediator expression in the interdigital space. TLR1 (A), TLR2 (B), TLR4 (C), TLR6 (D), IL-1β (E) and TNFα (F) transcript levels in clinically normal, mild ID (IDm), severe ID (IDs) and footrot (FR) biopsies of the skin/hoof interface. Data are presented as box and whisker plots showing minimum, lower quartile, median, upper quartile and maximum. Correlation of transcript expression of TLR2 with TLR4 (G) and IL-1β (H) for all samples.

**Supplementary Fig. S1:**
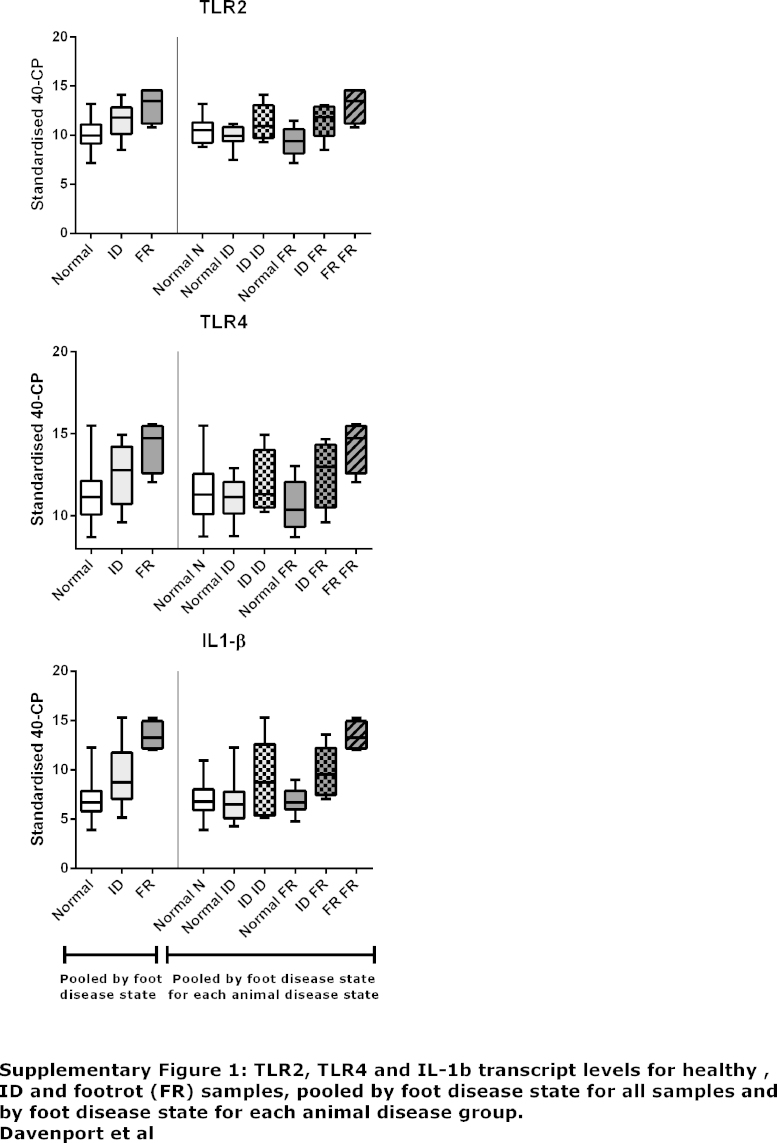
TLR2, TLR4 and IL-1β transcript levels for healthy, ID and footrot (FR) samples, pooled by foot disease state for all samples and by foot disease state for each animal disease group (healthy, ID, FR), whereby clinically healthy animals only had healthy feet, ID animals had healthy and ID feet and FR animals had healthy, ID and FR feet.

Expression of transcripts of TLR1, 2, 4, and 6 and the inflammatory mediators IL-1β and TNFα were analysed in biopsies from the skin/hoof interface of feet that were either clinically normal, had mild ID, severe ID or footrot (Fig. 1). Expression of *TLR2*, *TLR4* and *IL-1β* was significantly higher in severe ID (CI: TLR2 1.41–4.45, TLR4 1.41–4.8, IL-1β 1.35–6.27) and footrot (CI: TLR2 1.71–4.36, TLR4 4.65–4.63, IL-1β 4.37–8.68) (Fig. 1B, C and E). Expression levels were dependent on the disease state of the foot not the animal, i.e. in animals that had one or more foot affected with ID or footrot, the clinically normal feet had the same expression levels as normal feet from animals with no diseased feet (Fig. S1). In contrast, *TLR6* expression was lower in mild ID (CI: (−2.81) − (−0.22)) but not in severe ID and footrot (Fig. 1D). Expression of *TLR1* and *TNFα* was similar in all samples (Fig. 1A and F). A strong correlation between *TLR2* and *TLR4* expression was observed (Fig. 1G, *R*^2^ = 0.81). *TLR1* and *TLR6* expression did not mirror that of *TLR2*, suggesting a mixture of TLR1/TLR2 and TLR6/TLR2 heterodimers may be being expressed (Fig. 1A, B and D). However, *TLR6* was expressed at significantly higher levels than *TLR1* (*p* < 0.0001).

Supplementary figure related to this article found, in the online version, at http://dx.doi.org/10.1016/j.vetimm.2014.07.007.

### 3.2 ID and footrot lead to progressive pododermatitis with mixed neutrophil and lymphocyte infiltration

**Fig. 2:**
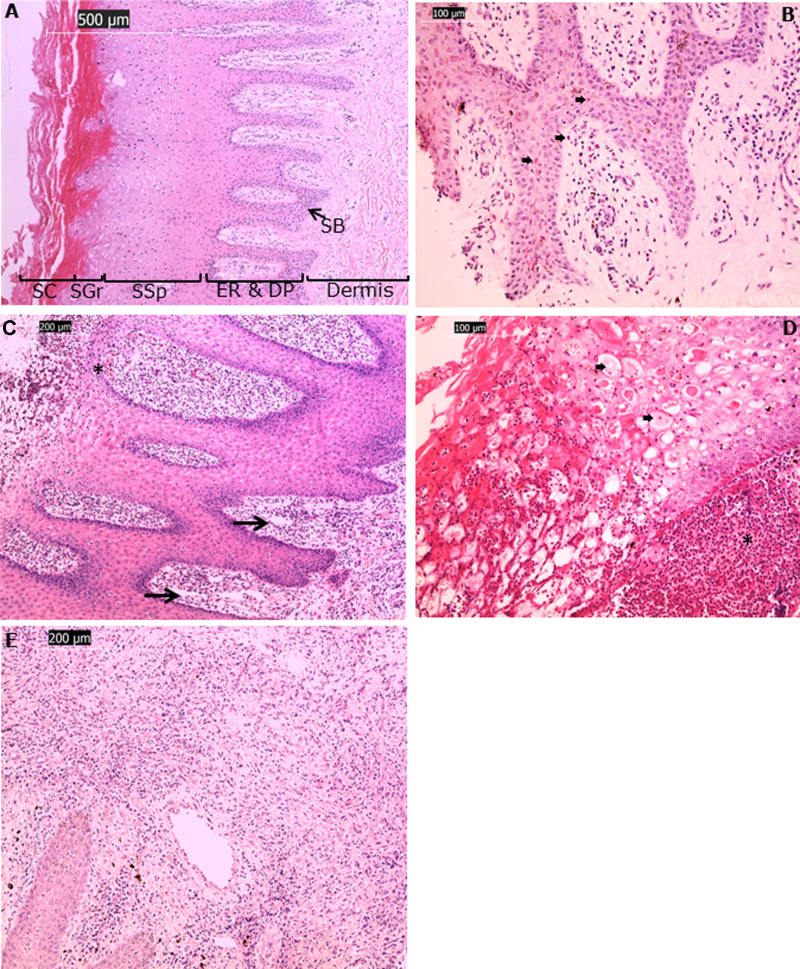
Histological features of clinically healthy, ID and footrot samples. H&E stain of clinically healthy (A): overview, with SC, stratum corneum; SGr, stratum granulosum; SSp, stratum spinosum; ER, epidermal ridges; DP, dermal papillae; SB, stratum basale; ID (B), superficial mild to moderate dermatitis, exocytosis, granulated neutrophils (); FR (C) marked dermatitis, oedema (arrow), congested vessels (*); FR (D) epidermal degeneration (ballooning with nuclear pyknosis) () and large numbers of degenerating neutrophils (purulence) (*) underneath the stratum basale; FR (E) dermal fibrosis.

Tissue sections from clinically normal feet had no obvious damage to the interdigital skin. The epidermis had a prominent granular layer and the dermal–epidermal junction had a low number of infiltrating leukocytes. The papillary dermis showed minimal to focally mild perivascular infiltration of lymphocytes and neutrophils (Fig. 2A). Thus, clinically normal feet had a minimal to mild chronic-active pododermatitis with a mixed lymphocytic and neutrophilic infiltration.

ID is characterised by erythema (redness of the skin) in the interdigital skin, which is a direct result of dermal blood vessel dilation and hyperaemia. Perivascular immune cell infiltration was seen surrounding dilated superficial dermal blood vessels and resulted in a cell dense dermal–epidermal junction, predominantly involving lymphocytes, neutrophils and a few plasma cells (Fig. 2B). Migrating leukocytes in the stratum basale and stratum spinosum represented cells that had moved through the dermal–epidermal junction (exocytosis) (Fig. 2B), with neutrophilic degranulation observed in the superficial epidermis. Overall, ID presented as a moderate chronic-active pododermatitis with a mixed lymphocytic and neutrophilic infiltration with mild epidermal exocytosis.

In footrot, in addition to erythema, under running of the hoof horn interface was observed indicating a loss of tissue integrity. The dermis showed large numbers of perivascular lymphocytes, neutrophils and fewer plasma cells leading to a cell dense migration through the papillary dermis and epidermis (Fig. 2C). Lymphocytes and neutrophils accumulated uniformly in all layers of the epidermis. Purulence, composed of large numbers of non-viable neutrophils, necrotic debris and plasma proteins, was seen frequently in areas of epidermal degeneration and necrosis and in areas of epidermal–dermal clefts (Fig. 2D). Epidermal ballooning, characteristic of hydropic cell degeneration, was noted with marked swelling of the cytoplasm and additional condensation (pyknosis) of the nucleus (Fig. 2D). Increased fibrous tissue proliferation (superficial dermal scarring) occurred in some cases indicating a chronic reaction due to the loss of tissue integrity at the skin hoof interface (Fig. 2E). The papillary dermis showed vascular congestion and dilated lymphatic vessels with dermal oedema (Fig. 2C), as a consequence of the intense epidermal and dermal inflammation. In summary, footrot presented as moderate to focally severe chronic-active pododermatitis with a mixed lymphocytic and neutrophilic infiltration, pus formation, horn clefting in some cases.

### 3.3 *TLR2*, *IL-1β*, *TNFα* but not *TLR4* expression is increased in dermal fibroblasts in response to *D. nodosus* and *F. necrophorum*

**Fig. 3:**
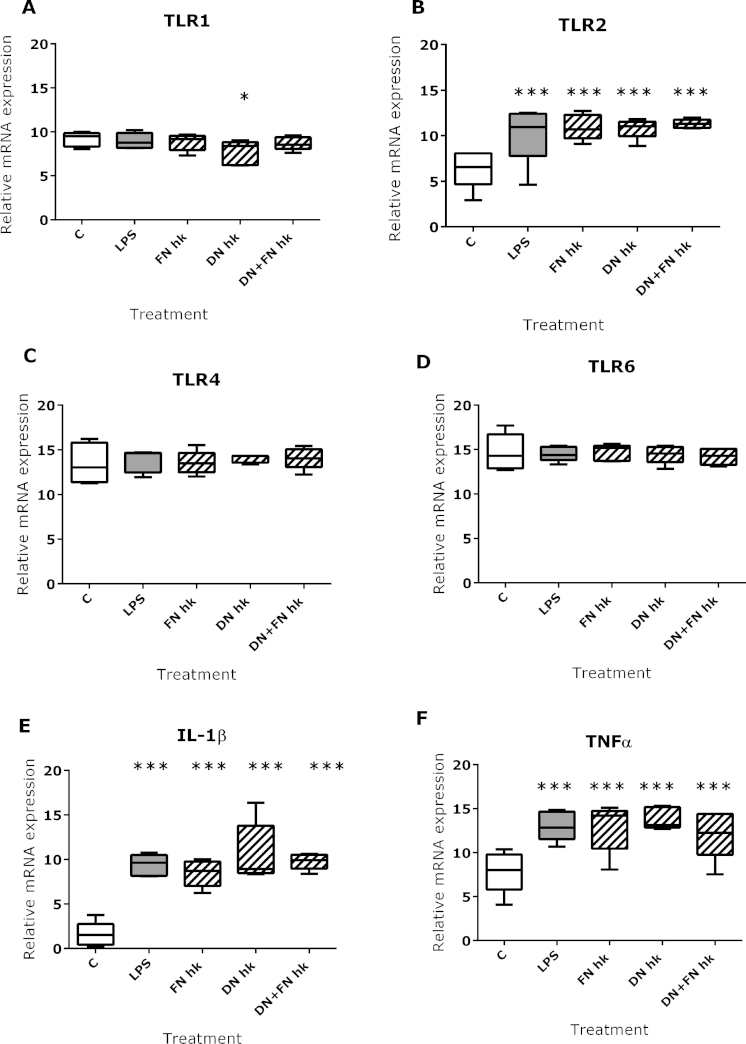
TLR and inflammatory mediator expression in ovine dermal fibroblasts. TLR1 (A), TLR2 (B), TLR4 (C), TLR6 (D), IL-1β (E) and TNFα (F) transcript levels in ovine dermal fibroblasts stimulated with 1 mg/ml LPS or 10 mg/ml heat killed *D. nodosus* (DN), *F. necrophorum* (FN) or both (DN + FN) for 4 h. Data are presented as box and whisker plots showing minimum, lower quartile, median, upper quartile and maximum. One-way ANOVA, repeated measures, Dunnett post-test, *p ≤ 0.05, ***p ≤ 0.001 to control.

Since bacteria were observed in the dermis in tissue sections from feet with footrot, we investigated *TLR1*, *TLR2*, *TLR4*, *TLR6*, *IL-1β* and *TNFα* expression in ovine dermal fibroblasts, a resident dermal cell type, in response to LPS and heat-killed extracts of *D. nodosus* and *F. necrophorum* (Fig. 3). In response to LPS and bacterial extract stimulation, *TLR2*, *IL-1β* and *TNFα* transcript expression was significantly increased in dermal fibroblasts (Fig. 3B, E and F); no effect was seen on *TLR4* and *TLR6* expression (Fig. 3C and D). *TLR1* transcript expression was unchanged except for a small but significant decrease in response to *D. nodosus* (Fig. 3A). As seen for the foot biopsies, *TLR1* and *TLR6* expression did not mirror that of TLR2, suggesting expression of *TLR1/TLR2* and *TLR6/TLR2* heterodimers. However, *TLR6* was expressed at significantly higher levels than *TLR1* (Fig. 3A, B and D, *p* < 0.0001).

## 4 Discussion

This study is the first to report changes in expression of TLRs and pro-inflammatory cytokines in ovine interdigital skin samples from clinically normal and feet diseased with ID or footrot. In interdigitial skin, *TLR2* and *TLR*4 transcripts were the most abundant, and higher levels of *TLR6* than *TLR1* were expressed, this is similar to clinically healthy ovine skin from the flank (Nalubamba et al., 2007). Both flank and interdigital skin are exposed to a range of commensal microorganisms, however, there is less potential for tissue damage due to environmental conditions in the flank area compared with the interdigital skin, where damage and subsequent opportunistic infection of pathogens or commensal skin flora are common. Enhanced expression of *TLR2* and *TLR4* in response to infection has also been observed in another ovine infectious disease, Johne's disease, caused by *Mycobacterium avium* subspecies *paratuberculosis* (MAP) (Taylor et al., 2008). TLR1 and TLR6 can both form heterodimers with TLR2, recognising bacterial triacyl lipopeptides and diacyl lipopeptides respectively (Farhat et al., 2008). In MAP infections, TLR2/6 heterodimers are involved in the recognition of mycobacterial antigen (Bulut et al., 2001, Gomes et al., 2008), and in MAP-infected ileum both *TLR2* and *TLR6* expression were significantly increased, supporting a role for this heterodimer in the pathogenesis of Johne's disease (Plain et al., 2010). In contrast, our study of severe ID and footrot did not result in an expression profile indicative of a similarly predominant TLR2/6 heterodimer. Since damaged interdigital skin is exposed to a diverse opportunistic skin and environmental flora with at least 27 bacterial genera (Calvo-Bado et al., 2011), having a more diverse array of pathogen receptors, such as both, TLR1/2 and TLR6/2 heterodimers, may be an advantage to the host. The ovine inflammatory response associated with ID and footrot is also reflected in the increased expression of the pro-inflammatory cytokine *IL-1β*. Interestingly, expression of *TLR2*, *TLR4* and *IL-1β* was dependent on the disease states of the individual feet of an animal. This suggests that the disease has a local focus in the foot with little or no systemic innate immune response. This is different from the humoral response, where foot scoring methods that summed up the lesions on all the feet and weighted underrun lesions relative to non-underrun lesions provided the most accurate association with serum antibody levels to *D. nodosus* (Whittington and Nicholls, 1995).

The results of our study illustrate that ID and footrot lead to progressive chronic-active pododermatitis with a mixed lymphocytic and neutrophilic infiltration. This is mild in clinically normal feet, moderate in feet with ID and focally severe in footrot, with frequent areas of purulence and lytic necrosis. The mild inflammatory phenotype seen in apparently healthy ovine feet is likely to be dependent on many factors, including the environmental conditions and the variability in levels of bacterial exposure in individual sheep and host resistance. Similar observations have been made in a previous study (Parsonson et al., 1967), apparently describing a mild ID, but with no comparison to the histological appearance of clinically normal interdigital skin. Hence it might be that clinically normal skin has a constantly active immune response due to repeated exposure to commensal and pathogenic microbes. The gross footrot lesions in this study were similar to those described previously with intense inflammation, necrotic tissue and separation of the hoof-horn interface throughout the whole interdigital space (Egerton et al., 1969).

In addition, the inflammatory response, seen at the transcript level, in severe ID and footrot potentially resulted in the infiltration of immune cells into the epidermis. Bacteria, including *D. nodosus*, were morphologically identified in both, epidermal and dermal layers of the skin (Egerton et al., 1969), hence it is important to understand the role that individual cellular components of the skin play in the pathogenesis of this disease. One of the main cell types of the dermis is the fibroblast, and although originally thought to only play a structural role in the skin, it has subsequently been shown that these cells have an important role in the innate immune response, providing the supporting extracellular matrix and, on activation, produce cytokines, chemokines and prostanoids (Buckley et al., 2001). The release of pro-inflammatory cytokines and chemokines such as IL-1β, IL-6 and CXCL8 is fundamental in the cross talk between the innate and adaptive immune systems, increasing diversity of responses and hopefully protection against re-infection (Buckley et al., 2001). Our results showed enhanced *TLR2*, *IL-1β* and *TNFα* expression in cultured dermal ovine fibroblasts in response to defined (LPS) and mixed bacterial ligands (heat-killed *D. nodosus* and *F. necrophorum*), which demonstrates the ability of dermal fibroblasts to mount a pro-inflammatory response, thus contributing to the recruitment of innate immune cells.

In summary, we present here the first study of innate immune responses in ovine ID and footrot, linking immunopathology and inflammatory mediator expression localised to the interdigital skin and dermal fibroblasts. The differential expression of TLRs and proinflammatory cytokines correlates with the disease state of the sheep foot and strongly demonstrates the importance of the local immune response with little or no impact of the systemic innate immune response. Hence understanding of those local responses, particularlythe role of TLRs, can contribute to developing different approaches to vaccination.
