# Supplementary material for: Differential expression of Toll-like receptors and inflammatory cytokines in ovine interdigital dermatitis and footrot
Source: Vet Immunol Immunopathol. 2014 Sep 15;161(1-2):90–8. doi: 10.1016/j.vetimm.2014.07.007 (PMC4157958; doi:10.1016/j.vetimm.2014.07.007)
Supplement: Supplementary Table S1 — Standard curve data from qPCR. [file mmc1.doc]

**Supplementary Table S1: Standard curve data from qPCR**

| **target** | **slope** | **R^2^** | **Efficiency (%)** |
| --- | --- | --- | --- |
| 18S | -3.262 | 0.999 | 103 |
| α-tubulin | -3.397 | 0.984 | 97 |
| β2-Microglobulin | -3.278 | 0.98 | 102 |
| PPIA | -3.111 | 0.981 | 110 |
| GAPDH | -3.814 | 0.967 | 83 |
| β-actin | -3.226 | 0.975 | 104 |
| TLR1 | -3.455 | 0.9938 | 95 |
| TLR2 | -3.3209 | 0.9919 | 100 |
| TLR4 | -3.4076 | 0.9922 | 97 |
| TLR6 | -3.339 | 0.9916 | 99 |
| IL-1b | -3.3819 | 0.9404 | 98 |
| TNFα | -3.3581 | 0.991 | 99 |

Efficiency = 10^(-1/slope)^ x 100%
